# Overcoming hurdles to intervention studies with autistic children with profound communication difficulties and their families

**DOI:** 10.1177/1362361321998916

**Published:** 2021-04-07

**Authors:** Ailbhe McKinney, Emma JL Weisblatt, Kathryn L Hotson, Zahra Bilal Ahmed, Claudia Dias, Dorit BenShalom, Juliet Foster, Suzanne Murphy, Sofía S Villar, Matthew K Belmonte

**Affiliations:** 1 Nottingham Trent University, UK; 2 The University of Edinburgh, UK; 3 University of Cambridge, UK; 4 Cambridgeshire and Peterborough NHS Foundation Trust, UK; 5 Ben Gurion University of the Negev, Israel; 6 King’s College London, UK; 7 University of Bedfordshire, UK; 8 The Com DEALL Trust, India

**Keywords:** autism, clinical trial, inclusion, intellectual disability, intervention, minimally verbal, nonverbal

## Abstract

Autistic children and adults who are non-verbal/minimally verbal or have an intellectual disability have often been excluded from Autism Spectrum Disorder research. Historical, practical and theoretical reasons for this exclusion continue to deter some researchers from work with this underserved population. We discuss why these reasons are neither convincing nor ethical, and provide strategies for dealing with practical issues. As part of a randomised controlled trial of an intervention for children with profound autism, we reflected as a multi-disciplinary team on what we had learnt from these children, their families and each other. We provide 10 strategies to overcome what appeared initially to be barriers to collecting data with this population. These hurdles and our solutions are organised by theme: interacting physically with children, how to play and test, navigating difficult behaviours, selecting suitable outcome measures, relating with parents, managing siblings, involving stakeholders, timing interactions, the clinician’s role in managing expectations, and recruitment. The aim of this article is to provide researchers with the tools to feel motivated to conduct research with children with profound autism and their families, a difficult but worthwhile endeavour. Many of these lessons also apply to conducting research with non-autistic children with intellectual disabilities.

**Lay abstract:**

Autistic children who speak few or no words or who have an intellectual disability are the most in need of new understandings and treatments, but the most often left out of the research that can bring these benefits. Researchers perceive difficulties around compliance with instructions, testing, challenging behaviours and family stress. Although research with these children can indeed be difficult, their continuing exclusion is unethical and unacceptable. Drawing on our experiences testing a possible treatment for children with profound autism, we provide 10 practical guidelines related to (1) interacting physically, (2) combining play and testing, (3) responding to challenging behaviour, (4) finding suitable tests, (5) relationships with parents, (6) relationships with siblings, (7) involving stakeholders, (8) planning the testing times, (9) the role of the clinical supervisor and (10) recruiting and retaining participants. We hope that these guidelines will prepare and embolden other research teams to work with profoundly autistic children, ending their historical exclusion from research. These guidelines also could be useful for conducting research with children with intellectual disabilities.

## Introduction

Children with profound autism may be non-verbal/minimally verbal, have an intellectual disability (ID), and/or severely impaired adaptive functioning with significant requirements for support (National Council on Severe Autism, 2019). Approximately, 30% of children with autism are non-verbal or minimally verbal (Tager-Flusberg & Kasari, 2013) and 33% have an ID (Maenner et al., 2020). Historically, this group has been largely excluded from autism research (Jack & Pelphrey, 2017; Russell et al., 2019; Tager-Flusberg & Kasari, 2013). However, the last decade has seen a growth of interest in research for autistic children with profound communication difficulties (henceforth, profound autism). Advocacy groups, especially in the United States, are pushing back against the paucity of research on profound autism, calling for the inclusion of the entire spectrum of Autism Spectrum Disorder (ASD) in research: the National Council on Severe Autism was established in the United States in 2019, the Autism Inpatient Collection (AIC; Siegel, 2018) in 2013 and the Strategic Plan for Autism Spectrum Disorder (Interagency Autism Coordinating Committee, 2020) was most recently updated this year. The 2018 annual meeting of the International Society for Autism Research debuted the Special Interest Group ‘Clinical Strategies for Including Severely Affected Individuals in Neuroscience Studies’. In the United Kingdom, Autistica, in response to its report (Warner et al., 2019) highlighting a lack of research for autistic people who have intellectual disabilities, set up the Complex Needs Autism Study Group which is funding projects such as ‘Including People with Complex Needs in Research’. The emphasis on autism without ID has spurred parent-led campaigns such as ABA Access4ALL (2016) to make interventions for profound autism more widely available.

Several research groups have made strides towards developing effective interventions for children who are non-verbal or minimally verbal (for full reviews, see Brignell et al., 2018; Koegel et al., 2019). Joint Attention Symbolic Play, Engagement and Regulation (JASPER), which has shown the effectiveness for toddlers with autism (Kasari et al., 2010), has also yielded therapeutic effects in school-aged children with autism who are minimally verbal. Using a sequential multiple assignment randomised trial (SMART), Kasari and colleagues (2014) showed improvements in spontaneous communicative utterances, novel words and comments in children who received the intervention combination of JASPER, Enhanced Milieu Teaching and the use of a speech generating device compared to those who received just JASPER and Enhanced Milieu Teaching. Further SMART studies testing adaptive interventions for children who are minimally verbal are underway (U.S. National Library of Medicine, 2013, 2020a, 2020b; NCT03883139, NCT04218331 and NCT01751698). Another comparison study found Auditory Motor Mapping Training combined with Speech Repetition Therapy evoked greater gains than Auditory Motor Mapping Training alone (Chenausky et al., 2016). A parent-mediated technique, Focused Playtime Intervention, showed improvements in expressive language for some children (Siller et al., 2013). Peer-mediated interventions for minimally verbal children have shown modest potential to improve social communication and expressive language, and to increase the use of augmentative and alternative communication (AAC), yet the majority of these studies have used single-case experimental designs and to date no comparison studies have been conducted to assay effectiveness (O’Donoghue et al., 2021).

Despite these strides, there remains no recommended intervention for this group, and large-scale multi-site randomised controlled trials (RCTs) have been few (Brignell et al., 2018; Koegel et al., 2019). These children’s difficulties, and interventions to remediate them, remain under-researched overall (Russell et al., 2019). The current set of guidelines aims to promote intervention studies and RCTs with this population.

Historically, the turn away from researching profound autism is related to the surge in research on ‘pure’ autism (without ID or other comorbidities) and the excitement around learning more about Asperger’s syndrome wherein circumscribed areas of interest, such as cognitive and sensorimotor processing and language, can quite easily be explored (Happé & Frith, 2020). Theoretical rationales can undergird researchers’ tendency not to include non-verbal persons, as they may wonder whether the potential comorbidity of another diagnosis (e.g. ID, Specific Language Impairment and attention-deficit hyperactivity disorder (ADHD)) might confound their results. To address this viewpoint, researchers tend to exclude the children with profound autism, although this blinkered sampling approach inevitably reduces the generalisability of results (Thurm et al., 2019). A more promising approach is to embrace complexity, recognising that finding a treatment for ‘pure’ autism does not bring a treatment for the whole spectrum: in a sample of 65 minimally verbal autistic children and adolescents, virtually every participant met criteria for at least one comorbid condition (Plesa Skwerer et al., 2019). Autistica (2019) recently launched the Embracing Complexity coalition of UK charities which highlighted that the majority of individuals with autism also have other neurodevelopmental disorders (e.g. ADHD and dyslexia), mental health problems, epilepsy and/or an ID.

There are practical reasons as to why researchers shy from therapeutic and other research with children with profound autism: (1) development of diagnostic measures appropriate to basic research has outpaced development of test–retest assessments sensitive to therapeutic change, which can in any case be difficult to adapt for a population who have trouble following verbal, often sequential instructions (Stedman et al., 2019); (2) challenging behaviours (Tager-Flusberg et al., 2017) can be difficult to manage and could be especially off-putting to early career or non-clinical researchers; (3) parents of children with profound autism tend to be under more stress than parents of autistic children with lesser support needs or typically developing children (Ingersoll & Hambrick, 2011; Rivard et al., 2014) and struggle to find energy and time to participate in research, although they are aware that it is needed (indeed, in our recruitment process and retention rates, stress has been a reason parents provided for not taking part, cancelling appointments or dropping out); (4) ascertaining assent from children and adults with profound autism or ID is difficult; and (5) early-career researchers (e.g. PhD students/post-doctoral researchers), non-clinical autism researchers (e.g. cognitive neuroscientists) or clinicians new to research may not feel equipped with the skills to develop and/or test hypotheses or interventions for this population. In sum, it is perceived to be harder to conduct research with autistic children with more complex needs.

This issue is beginning to be addressed by the research community with regard to lab-based experiments. Kylliäinen et al. (2014) provide useful guidelines for conducting research with children with profound ASD using electroencephalogram (EEG), magnetoencephalography (MEG), eye-tracking and measurements of heart rate and skin conductance. They discuss stimulus selection, experimental set-up, a desensitising period for wearing a cap or helmet, as well as more general guidelines including duration of the experiment, making use of parents’ know-how and the types of feedback to give to parents. Nordahl et al. (2016) have successfully collected magnetic resonance imaging (MRI) data with school-aged children with autism and an intellectual impairment, without sedation, by employing principles of Applied Behaviour Analysis. Similarly, Tager-Flusberg et al. (2017) provide advice for conducting research with minimally verbal children using behavioural, eye-tracking and EEG techniques. They note challenging behaviour as one of the main hurdles and suggest solutions such as redirecting the child’s attention, changing the activity, offering a short break or ignoring outbursts.

In this article, we focus on collecting behavioural data in naturalistic environments rather than in the lab, and on strategies specifically relevant to conducting a clinical trial with children with profound autism and their families through clinical services. While interventions for children with profound autism are in development, a concise and accessible set of guidelines for conducting an intervention study with this population does not exist to our knowledge. It is our hope that this article can grease the wheels, hastening the goal of developing effective interventions. While experienced clinical researchers and clinicians may already be aware of many of these principles, a large proportion of data collection and interaction with families and children is carried out by students, early career and non-clinical researchers, at whom this article is aimed.

### Aim

In sharing general insights based on our own experiences, we provide strategies for collecting high-quality data and conducting research confidently with autistic children with profound communication difficulties. We hope this article will widen the participation from autism researchers and guide good practice for groups who are developing an intervention study.

## Method

### Participants

We report on the lessons from recruiting and testing 30 non-verbal or minimally verbal children with autism for our Point OutWords intervention feasibility RCT (trial registration; ISRCTN12808402, protocol: McKinney et al., 2020, website: http://PointOutWords.online/). This article is separate from our main feasibility results paper, forthcoming. In brief, then: children were randomised to receive our iPad app Point OutWords or a selection of control apps. Children and parents were tested at baseline and after an 8-week intervention period. The protocol was approved by the UK National Health Service (NHS) Nottingham 2 Research Ethics Committee, and written informed consent was obtained from all participating families. The data for this trial were collected by one main researcher (the first author, AMK) who was always accompanied by one of the four research assistants. Although safeguarding demanded the presence of two researchers during testing, in practice it was found in any case to be a two-person job. Specific tasks allotted to the primary and the secondary researcher will be discussed.

We recruited through four NHS trusts in Peterborough, Kettering, Sheffield and Nottingham, England. Table 1 summarises participants’ ages, genders and ethnicities by NHS trust. As this was a feasibility trial, baseline measures were not used to screen participants; instead, clinicians screened based on their clinical judgement and clinical records. Clinicians were asked to recruit children with an autism diagnosis who could understand more than they can say and who had motor impairments. We defined non-verbal as speaking no words and minimally verbal as speaking fewer than 100 words with no phrase speech (see Table 2 for parent-reported descriptive information about the sample). Specific data on socioeconomic status were not recorded; however, the clinic from which the majority of participants were recruited is located in an area of predominantly low socioeconomic status.

**Table 1.** Participants’ ages, genders and demographics.

NHS trust	Cambridgeshire and Peterborough Foundation Trust	Northamptonshire Healthcare NHS Foundation Trust	Nottingham University Hospitals NHS Trust	Sheffield Children’s NHS Foundation Trust
Location	Peterborough	Kettering	Nottingham	Sheffield
	(*N* = 22)	(*N* = 4)	(*N* = 3)	(*N* = 1)
Age at pre-testing (years)				
Range	3.33–15.67	4.5–15.83	7.25–15.58	NA
Mean (SD)	7.13 (3.47)	10.79 (4.69)	12.31 (4.44)	2.92
Race/ethnicity				
White British	11	1	3	1
Mixed British	2	1	0	0
Other White	3	1	0	0
Asian	1	1	0	0
Mixed South Asian/British	2	0	0	0
Other mixed	1	0	0	0
African	1	0	0	0
Unknown	1	0	0	0
Sex				
Male/female	15/7	3/1	3/0	1/0

NHS: National Health Service; SD: standard deviation; NA: not available.

**Table 2.** Age equivalent baseline scores in years on the Vineland Adaptive Behavior Scales (VABS-II).

	Mean (SD)
Communication	
Receptive	1.33 (0.82)
Expressive	1.3 (0.62)
Daily living skills	
Personal	2.09 (1.03)
Domestic	2 (1.19)
Community	2.37 (1.02)
Socialisation	
Interpersonal	0.84 (0.6)
Play and leisure	1.2 (0.53)
Coping skills	2.5 (0.58)
Motor skills	
Gross motor skills	3.56 (1.275)
Fine motor skills	2.58 (1.42)

SD: standard deviation.

### Reflective process

With the view to assess and improve the feasibility of the clinical trial, the main research assistant kept a diary throughout data collection. She recorded experiences and reflections over an 11-month data collection period from April 2019 to March 2020, often reflecting with the other research assistants. This iterative reflective process was facilitated by discussions with members of the research team, in particular the clinical Chief Investigator (CI) (EJLW) and the patient and public involvement (PPI) coordinator (CD). Challenges and solutions are organised by theme below, extracted by the process of reflection, discussion and reporting.

### Community involvement

The Point OutWords app and the intervention design were co-produced with children with profound autism, their parents and their therapists (Weisblatt et al., 2019). The stakeholder coordinator on our research team was a mother of two children with profound autism. We reflect on our Patient Involvement in more detail in section ‘Lesson 8: stakeholder involvement is intrinsic to every part of a project’.

## Results

### Lesson 1: physical interaction can facilitate testing

Sensory issues are one of the first practical hurdles to overcome when testing children with autism as such differences are almost universal in the form of sensitivities or sensory seeking (Patten et al., 2013; Tavassoli et al., 2019). When selecting stimuli and outcome measures, it is essential to consider this constraint (Balasco et al., 2020; Kylliäinen et al., 2014). Yet, caution around tactile sensitivity, for example, may lead researchers new to autism to think that they cannot touch an autistic child at all, for fear that doing so might evoke challenging behaviour or might disturb the testing relationship between the child and researcher. Indeed, a supposed dislike of touch was recently highlighted as a ‘myth’ about autism (John et al., 2018). It has been this research team’s experience that encouraging children and facilitating testing through touch are essential to completing data collection, especially in a population of children with profound autism, where communication abilities are most impaired. Tactile or other simple sensory prompting can be much more salient than conventional social cueing, and correspondingly more effective at redirecting or maintaining attention for this population (Chen et al., 2012). For example, patting a child on the shoulder for praise, leading them to the testing area by taking their hand or placing their hand on a testing object is perhaps the most accessible means of communication. Furthermore, playing sensory games during testing may relax the child and help build a rapport, for example, rolling a car up and down their arm. While sensory needs are essential to consider, tactile stimulation is not to be totally avoided and instead, with parental permission and guidance, should be viewed as a means of facilitating testing.

### Lesson 2: how to play and how to test: the child should not know the difference

The researchers should present themselves as someone there to play with the child. When we arrived at the home or the family arrived at the healthcare setting, we found it helpful to start playing with the child immediately. One research assistant can interact with the child while the other obtains consent from the parent and explains the testing procedure. Our testing suitcase was brightly coloured and bore many tactile stickers of animals, providing high chromatic and luminance contrast at a range of spatial frequencies that tended to outcompete the surrounding environment; it was highly salient, without being painfully so. We found that most children gravitated towards it, and naming the animals or making the animal sounds often served as an ice-breaker. With attention thus captured by the suitcase, the research assistant could open it and take out materials to transition smoothly into the first test. Koegel et al. (2020) mention the importance of naturalistic settings for testing children who speak few or no words. We gave parents the option for us to go to their house or for them to come to a healthcare setting. Parents were asked to have toys with which the children usually play.

Testing should be integrated in between playing, and the researcher should aim to move flexibly, swiftly and seamlessly between play and testing. When the child’s attention wanders and they start playing with something else, it is advisable to be led by the child, joining in this play with whatever has grabbed their attention. This way, the researcher and child remain ‘locked’ in joint play and the researcher maintains themself in the state of relevance necessary to redirect attention back to the assessment. The aim is to strike a balance between letting the child lead and guiding the child to the next activity. The researcher should not take it personally if the child does not seem to like them or to want to play at first. In fact, this scenario is common– the researcher is an unknown adult, often intruding in the child’s own home. The researcher should continue to attempt to develop a rapport. After a few attempts, the secondary research assistant can try. If that still does not work, the researcher can take guidance from the parents about whether this behaviour is usual for the child and what helps in similar situations. In most cases, however, a rapport with the child is achievable.

We also found it important to understand that developmental trajectory in autism can be non-linear and domain-specific. A child with autism may not speak but still be able to count blocks, draw shapes or complete other tasks that are further along developmental assessments normed with typically developing children. Researchers thus should not assume a child’s developmental ability in one domain (e.g. motor skills) based on another domain (e.g. expressive language). Furthermore, it is important that the research assistants maintain high expectations of the child’s ability throughout testing as this will facilitate the child’s reaching their full testing potential.

### Lesson 3: using suitable measures

The need for suitable and accurate outcome measures for children with profound autism is beginning to be acknowledged and addressed in the literature (Kasari et al., 2014; Plesa Skwerer et al., 2016; Trembath et al., 2019; Trembath & Iacono, 2016). There is a dearth of standardised assessments specifically for children who are minimally verbal and so research groups tend to adopt standardised developmental assessments. In line with the recommendations of Kasari et al. (2014), we used a combination of standardised and experimental measures. Table 3 lists the parent and child standardised measures and the coding frameworks used to score 12-min videos of naturalistic parent and child play. We also collected data directly from the iPads, measuring kinematics of all interactions with the iPad. The two novel aspects of this selection of outcome measures were the kinematic data acquired through the intervention itself, and an oral motor assessment which had not been tested on this population before.

**Table 3.** Outcome measures.

Assessment name	Variable assessed	Child vs parent	Citation
Mullen Scales of Early Learning	Expressive language Receptive language Fine motor skills	Child assessment	Mullen (1995)
Verbal Motor Production Assessment for Children (VMPAC)	Neuromotor integrity of the speech system	Child assessment	Hayden & Square (1999)
British Picture Vocabulary Scale III (BPVS-III)	Receptive vocabulary	Child assessment	Dunn et al. (2009)
Brief Observation of Social Communication Change (BOSCC)	Autistic symptoms	Child assessment (coded from video of parent–child play)	Grzadzinski et al. (2016)
Dyadic Communication Measure for Autism (DCMA)	Communication interaction between parent and child	Child assessment (coded from video of parent–child play)	Aldred et al. (2012)
Vineland Adaptive Behavior Scales II (VABS-II)	Adaptive behaviour	Parent report	Sparrow et al. (2005)
Parenting Stress Index IV (PSI-4)	Stress in the child–parent dyad	Parent report	Abidin (2012)
Family Quality of Life Survey (FQoL)	Satisfaction with quality of family life	Parent report	Hoffman et al. (2006)
Autism Impact Measure (AIM)	Core autism symptoms	Parent report	Kanne et al. (2014)
Social Responsiveness Scale II (SRS-2)	Communicative competence	Parent report	Constantino & Gruber (2012)

Trembath et al. (2019) highlight the potential of innovative technologies to improve assessment but point out the need for accessibility. In the case of our iPad-assisted intervention, while the child enjoys completing puzzles with their parent, the iPad logs the child’s vocal responses and the temporal and spatial trajectory of every contact with the touchscreen, whence can be derived measures such as acceleration and visuomotor targeting error. In other interventions, such motor measures are picked up by smartphones or body-mounted sensors (Goodwin et al., 2019). Other groups developing app-assisted interventions should consider this novel approach, designing the app itself to collect data during the intervention.

Koegel et al. (2020) recommend a speech production assessment in autism studies. Kasari et al. (2014) note the lack of appropriate speech sound production assessments for children with autism who are minimally verbal. We tested the Verbal Motor Production Assessment for Children (Hayden & Square, 1999), which to our knowledge has not been used with this population before. Domains involving instructions or imitation of the researcher were not acceptable to many children, possibly because they did not strongly resemble the activities that a child would undertake during play. Whether a child completed an item, for example, ‘stick out your tongue’ was more reflective of their understanding of the instruction or ability to imitate than their oral motor control. However, domains which were based on observation, for example, *is chewing coordinated* were useful. It is advisable to use tests that feel as much as possible like a game to the child, with limited instructions. One such alternative is the Com DEALL Oro Motor Assessment (Archana, 2008), which permits more naturalistic but time-consuming observation and whose ‘speech movements’ and ‘tongue movements’ subscales correlate with the Verbal Motor Production Assessment for Children (VMPAC) but yield a greater dynamic range within an autistic sample, while placing typically developing children at or near ceiling. We likewise attribute our project’s success with the Mullen Scales of Early Learning (Mullen, 1995) to the Mullen’s ability to exploit play activities to assess fine motor skills, for example, drawing or stacking blocks. Similarly, the British Picture Vocabulary Scale’s (Dunn et al., 2009) bright colours and attractive pictures were appropriate for most of our sample who could achieve the praxis required to follow verbal instructions such as ‘point to the cat’. Plesa Skwerer et al. (2016) found similarly that most, although not all, children who are minimally verbal understand a vocabulary task that demands object-based pointing in the context of the Peabody Picture Vocabulary Test-4 (Dunn & Dunn, 2007).

As part of the feasibility assessment, we are analysing the suitability and acceptability of candidate measures to produce predictive information at baseline and to assess sensitivity to change over time, including the Autism Impact Measure (AIM; Kanne et al., 2014) – a new measure developed for autism treatment studies which has not been tested on this population before. For more information on the rationale behind the selection of each measure, see our protocol (McKinney et al., 2020).

### Lesson 4: challenging behaviour and consent

Tager-Flusberg et al. (2017) note challenging behaviour as a cause of difficulty collecting data with this population. Challenging behaviours can include perceived aggression, self-injury, socially inappropriate behaviours (spitting, exposure and inappropriate touching) or absconding. Researchers can learn from teachers and therapists who routinely recognise that all behaviours are communicative (Donnellan et al., 1988, 1984) especially in the case of those children who do not use speech to communicate. This appreciation of the communicative function of behaviours (Donnellan, 1984) demands their construal as acts of meaning rather than as a ‘problem’ for the research team. We experienced most of these behaviours, as well as crying, shouting and aggression towards the parent. It was our experience that such moments were often fleeting and for the most part could be resolved by giving the child a break, distracting them with a toy or a YouTube video, giving them a treat or praising them for their good work. These tactics often were enough to end the behaviour and to allow further engagement. Thus, although such behaviours can potentially be unfamiliar or worrying for early career or non-clinical researchers, we stress that they are more a hurdle than a barrier to research. The presence of two researchers during testing allowed extra support if the child became unsettled. Researchers can take context and advice from parents, and also from the team’s stakeholders and clinicians.

Perceived aggressive or other behaviours making it hard to complete testing can be related to the child’s mood, how much they slept, what they have eaten or whether they are feeling sick. In a population with little or no verbal communication, behaviour may be the only means that the child has to say ‘no’ which makes it highly relevant to the issue of assent and consent. Assent to take part is an ongoing process where engagement/disengagement indicates the presence or absence of assent to participate. Knocking over a vocabulary board might mean, among other things ‘I do not want to do that, can we do something else?’ The research assistant can use their clinical judgement and parental guidance to decide whether it is best to have a break or to come back another day. Similarly, self-injurious behaviour (hand biting and hitting oneself with a toy) could be, inter alia, an indicator that the child is anxious and it might be time for a break. However, it also may be a sensory behaviour frequently carried out by that child and might actually help them complete the task. Asking parents if they are concerned and being led by them is essential. Researchers should keep records of any behaviours that concerned them and discuss these in supervision.

### Lesson 5: maintaining relationships with parents and using parental guidance during testing

To manage expectations, the researcher must be transparent about not knowing whether the experimental treatment will be effective. Also, with a view to maintaining good relationships with parents, we told them they would receive treatments A and B, and that if they got A first they would get B after and vice versa, as opposed to using language with passive connotations such as ‘control group’. It is important to remember to praise parents and their child throughout the study. Such positive feedback, well deserved in any case as people give freely of their time and energy to carry out studies, also helps to maintain motivation to take part. It is important to confirm with parents that they should be proud of their family for taking part, as they are potentially helping other families like theirs in the future. It is important to comment on how well the testing procedure went and how lovely their child is to play with, as opposed to the child’s performance on tests. Along the same lines, some parents asked for advice like ‘When will my child talk?’ or ‘Will my child work when they are older?’; such questions are every parent’s concern but arise especially readily in cultures or communities where individuals are expected to fit into socially defined norms and roles (Daley, 2004), and professionals are regarded as comprehensive and unquestionable authorities whose job it is to fix the problem. Researchers responded that they were not qualified to answer those questions and referred them to the clinical lead.

This theme is central to all our experiences. During testing, parents’ expert knowledge of their child is invaluable in two ways. First, they can indicate if their child’s behaviour is usual, and make an informed decision about whether it would be worth coming back another day or if their child will not be able to participate. Second, the parent can facilitate testing by administering some items themselves or demonstrating the task for the child, as well as interacting with the child and supporting the researchers to understand their child. While talking to the parents is the most valuable means of predicting whether a child will comply with testing procedures, it is also important to point out that we found a number of parents were pleasantly surprised at their child’s abilities as demonstrated during testing. Kylliäinen et al. (2014) report a similar experience. While being guided by parents, the researcher must be careful not to let parents’ expectations lead to underestimation of a child’s abilities.

### Lesson 6: siblings are a part of testing

Siblings of the children taking part in the study were generally present at testing at the family home or in the clinic, and we found that usually they wanted to be included. 18-month-old toddlers would scream from prams and 16-year-old teenagers would sheepishly linger in the living-room doorframe, both hoping to be acknowledged and included. The researcher has the ethical responsibility to acknowledge them. Siblings could pose a challenge to testing sessions by taking the attention of the researcher, the parent or the participant. However, the researchers are in their space and making demands on them too, constraining their activities such as TV time or spending time in a particular room. Although we could not have predicted it at the start of this project, the secondary researcher’s main job sometimes became occupying the siblings. For this reason, we found it advisable to have toys and books in the bag suitable for older children and children without disabilities. The primary researcher collected the data and the secondary researcher passed the toys or testing materials to the primary researcher, supported the primary researcher and played with the siblings. These roles were interchangeable depending on whether a researcher felt more confident with a particular test or if a researcher felt that they had made a bond with the participant already. While it would be easier at times if the siblings were not present at testing, such an arrangement was not an option for most parents. We recommend the above strategies to those collecting data, particularly in the home: researchers should prepare for the likely prospect that siblings will be present during data collection and have a contingency plan in place to occupy them.

### Lesson 7: timing is everything

Motivation and energy to complete tasks are a limited resource for children, parents and researchers. Kylliäinen et al. (2014) report a range for how long a child can concentrate and sit still for an uninterrupted period from 20–30 min for a 7-year-old without ID to 2–10 min for a 3-year-old with profound autism. The researcher must thus constantly monitor the child’s interest and energy levels. It was helpful for the secondary researcher to pass toys and materials to the researcher collecting the data, and also to remove the testing materials no longer needed so as to avoid distraction or perseveration. Play breaks, toilet breaks or snack breaks were offered. We did not plan a time for breaks but rather responded with a break whenever there was a sudden drop in the child’s engagement.

If a child has been engaged for 40 min to an hour and now will not engage with the researcher and the task, it is important to recognise that they are tired and not to push the child any further. The researcher who pushes a fatigued child to finish the last few items on a test risks spoiling not only the test data but also the relationship with the child and possibly the parent. The researcher should use their clinical judgement to know when to come back another day or to accept that they cannot complete every task on the list. With the expectation that this issue of fatigue will render some data sets only partially complete, the order of test administration should be fixed so that the most essential data are collected earliest. In our experience, parents tend not to post questionnaires back when asked. Instead, a research assistant can offer to play with their child while parents fill them out, suggest that the parents fill them out in time for the next visit or offer to collect them by hand.

In planning a project, we found that it was advisable to allocate extra testing time for the inevitable cancellation of visits. Approximately one-fourth of all our scheduled appointments were cancelled for the following reasons: participant is sick, sibling is sick, participant is in a bad mood, parent forgot about the appointment and parent is dealing with other family/work problems. For example, if a researcher estimates that pre-testing three children will take nine sessions, they should assume that a quarter will be cancelled and allocate time for twelve testing sessions. Researchers should keep in mind that parents’ schedules might not be able to accommodate picking up again with testing the following day. Therefore, if an appointment is cancelled it can add a week to pre-testing, not just one day. It is advisable to send a text-message reminder the day before to confirm the visit. We found that it was good for the team’s morale to book two or three visits with different families in a day, so that if one was cancelled the whole day would not be a disappointment.

### Lesson 8: stakeholder involvement is intrinsic to every part of a project

Explicit strategy for PPI is rightly becoming a key requirement in applications for research funding (Staniszewska et al., 2018). Furthermore, trials that have a PPI component enjoy heightened rates of recruitment and retention (Crocker et al., 2018). The Point OutWords app and the intervention design were co-produced with children with profound autism, their parents and their therapists (Weisblatt et al., 2019). The stakeholder coordinator on our research team was a mother of two children with profound autism (CD). She led the team in the co-production of materials (e.g. instruction booklets, letters and message to parents and survey questions), advised research assistants on sensitivity to families’ expectations and needs and was available to families to consult directly (particularly, at the onset of the COVID-19 pandemic of 2020).

Our PPI coordinator also helped the whole team to place the study in the broader context of families, communities and lived experiences: As children grow older, parents’ optimism towards services and research fades. Parents often do not feel supported by clinical services, and therefore may feel disinclined to participate in research associated with these same services. Researchers should have an awareness around the contacts within, and interventions offered by, clinical services as parents might want to ask questions.

It is likely that our PPI co-production approach led to higher recruitment and retention. Still, some parents declined participation and many reported that administration of our large number of candidate test–retest outcome measures – typical in a feasibility study – posed a burden. Most parents reported that they enjoyed their participation and the accompanying sense of community. Clinical research teams can capitalise on this sense of community by building a visible presence of the research team in the clinical services, events, and social and other media. Histories of negative experiences with clinical services are common in this group, and clinical and research teams must exemplify a counterpoint to these. Doing so can be as simple as listening: Families with a child with autism are often excluded within the community and can become socially isolated (Kinnear et al., 2016; Mitter et al., 2019). Researchers may be the only adults with whom parents are able to talk about their child, or the only adult to take time to listen at all. As well as telling parents about the study, researchers must listen to what parents have to say. Building stakeholders’ positive perceptions of research depends on involving them as partners to create positive experiences.

Research with this communication-impaired population often demands that patients’ involvement be mediated by persons – usually family members – who know well the population in question and can voice their interests and concerns, as a supplement to assent from patients themselves. Qualitative data from parents (collected via diaries, feedback sheets and an online survey) complement quantitative results in assessing and improving feasibility. Basic researchers can be unaccustomed to the notion of a clinical feasibility study, which goes beyond piloting an experiment. A feasibility study, involving and partnering with a small sample of stakeholders to assess processes, measures and techniques, of which only a subset might be carried forward to a full-scale RCT, is the surest way that a researcher can be confident about whether the study that they have in mind will be acceptable to children with autism and their families (Kylliäinen et al., 2014): this point is especially important where observation and engagement are the means of determining acceptability in a group with profound communication difficulties.

### Lesson 9: the clinical CI’s role in supporting the research assistants and maintaining morale

The last two lessons are particularly relevant for the clinical lead on a multi-disciplinary team (MDT). The role of the clinical CI is officially, in addition to their scientific role, one of the managing clinical and safeguarding risks and clinical and information governance, as well as liaising with clinical services for recruitment. Another important role emerged during the project: maintaining the morale and supporting the training and development of the non-clinical and relatively inexperienced research assistants. At the outset, the CI explained to the research assistants a number of issues to expect, namely, that families would drop out, want to talk about difficult topics and become upset, and that children would vary widely in how they managed sessions from one day to the next and in their ability to complete tests at all. Perhaps predictably, this caution was not fully absorbed by the research assistants until later into the project, and setting up regular supervisions from the start would have been helpful to support research assistants to reflect and to understand that they were not to blame for dropouts, cancellations or parents’ being stressed. More regular supervision was introduced as the study continued. It was also useful for research assistants to sit in beforehand on a clinic with a non-verbal child with autism, to gain experience of sitting with and interacting with a child and family while not having a research task to complete. Overall as well as more formal roles, hitting the balance between managing overly optimistic expectations, while not being negative about what was achieved, is an important role of the clinical CI.

### Lesson 10: best strategies for recruitment

The traditional method of reviewing all records of autistic patients seen in the service over the past 10 or so years and writing to parents did result in successful recruitment, but at a slow rate. Many patients with clinical records had moved city, had telephone numbers listed that were not recognised, or did not pick up the phone or call back. Other reasons for not taking part included parents’ stating that they were too busy or not interested. Asking MDT members to recruit participants was also not successful, even after several visits to MDT meetings and provision of recruitment materials to colleagues – clinicians making diagnoses in busy clinics are not able to prioritise also asking parents about a research project, however, supportive of the project they may feel. The most effective strategy proved to be the clinical CI’s reviewing the records of the young children currently open to the service, either when the team were providing feedback just after diagnosis or during parents’ more optimistic stage within months of diagnosis, and writing to them directly herself. Per Lesson 8 above, one of the most effective recruiting tools is the involvement of one or more stakeholders on the research team, who can communicate the study to families who may have had negative prior experiences with research.

Researchers planning to recruit from this non-verbal or minimally verbal population should plan on a lower recruitment rate than they might expect on the basis of their or others’ experience with less profoundly affected individuals, and on a correspondingly wider net covering multiple clinics or sites. To work with other clinical sites most effectively, a multi-site approach needs to be set up from the start, and significant time must be available for the clinical CI to visit and have ongoing liaison with recruiting clinicians at those sites.

## Discussion

Children with learning difficulties have poor educational, mental and physical health outcomes and they and their families can become increasingly marginalised from society (Rickard & Donkin, 2018). An anecdote from our recruitment period illustrates this point: one mother thought that the research team rang her by accident. Jadedly she said, ‘no, my son does not talk; it will not work for him’. She was surprised when we explained that the intervention was specially designed for children who did not talk or said only a few words.

It is not surprising some parents feel this way as there are no current routinely recommended interventions for non-verbal or minimally verbal autistic children (Koegel et al., 2019), with the exception of the Pre-school Autism Communication Trial therapy (Pickles et al., 2016) and Applied Behaviour Analysis, but these are not widely available in the United Kingdom. A lack of support from services in many communities and a dearth of appropriate interventions send the message to parents that their child cannot be helped. As with any other disempowered population, these feelings become internalised and families may become increasingly hopeless and isolated. Parents often feel judged by society, resulting in feelings of guilt, incompetence and embarrassment (Ludlow et al., 2012). It is a relief when someone understands, helps parents without judgement and expresses some positive expectations of the child. An important role for researchers is to empower parents by encouraging their efforts and praising the child and parents for completing testing.

Completing an intervention programme along with pre- and post-testing is not feasible for some families who have a child severely affected by autism. The battles with education, services and sometimes their own extended family take a toll on parents’ health and well-being. However, dropout is inevitable in all trials and higher rates with this group do not mean that research with this population as a whole is not feasible. In conducting this clinical trial, we met with several apparent barriers, yet as a team, working with and learning from the families who took part, we have developed strategies for overcoming them which will facilitate our and others’ future research. It is important to have in place protocols to handle contingencies such as challenging behaviours, parents’ requests for advice or prognostication and safeguarding.

Our parent-delivered, iPad-assisted intervention Point OutWords aims to train prerequisite or ‘back door’ skills for communication development (Karanth et al., 2010), that is, fine and oral motor skills and understanding of symbolic representation. Within our heterogeneous sample, we also aim to evaluate for whom the intervention might be most effective, in terms of baseline measures and characteristics (McKinney et al., 2020). Such moderating characteristics should be evaluated in intervention studies as no single intervention will be suitable for all children with profound autism. This study also aims to evaluate any concomitant improvements in family quality of life and parent stress – research questions beyond direct effects on the individual which can be all too easily overlooked.

In addition to these practical guidelines which we hope will serve to improve the design and the day-to-day running of the clinical trial, large sample sizes, multi-site designs, the inclusion of treatment-as-usual groups (as opposed to comparing to another intervention) and the use measures of well-being and quality of life will raise the standard of intervention studies for children with profound autism (Brignell et al., 2018). Children affected by profound autism and their families are in greatest need of help and intervention, yet remain under-represented in clinical trials and in research more generally. Including children with profound autism and their families in more research most importantly holds the potential to increase equity and improve outcomes for this underserved group, but also will deepen the research community’s understanding of the disorder (Siegel, 2018). It is our human duty to avoid replaying the marginalisation and exclusion experienced by people with profound autism and their families, and we hope these guidelines and strategies will empower and encourage more researchers to do so.
